# Genomic sequencing of multicystic mesothelioma finds cohesin complex mutations associated with disease recurrence in patients referred for cytoreductive surgery and HIPEC

**DOI:** 10.1038/s41416-026-03366-5

**Published:** 2026-03-14

**Authors:** Jane Gibson, Norman John Carr, Sophia Stanford, Amatta Mirandari, Thomas Desmond Cecil, Reuben J. Pengelly, Steven Turner, Jonathan W. Essex, Konstantinos Boukas, Kevin Hocking, Manuel Dominguez, Faheez Mohamed, Sanjeev Paul Dayal, Alexios Tzivanakis, Brendan John Moran, Gbadebo Adeleke, Alex Mirnezami, Sarah Ennis

**Affiliations:** 1 Cancer Sciences, Faculty of Medicine, University of Southampton, Southampton, UK; 2 Bio-R Bioinformatics Research Facility, Faculty of Medicine, University of Southampton, Southampton, UK; 3 Human Development and Health, Faculty of Medicine, University of Southampton, Southampton, UK; 4 Peritoneal Malignancy Institute, Basingstoke and North Hampshire Hospital, Aldermaston Road, Basingstoke, Hampshire, UK; 5 School of Chemistry and Chemical Engineering, University of Southampton, Southampton, UK; 6 Wessex Investigational Sciences Hub, Cancer Sciences, Faculty of Medicine, University of Southampton, Southampton General Hospital, Southampton, UK; 7 Wessex Genomics Laboratory Service, Salisbury District Hospital, Salisbury, UK

**Keywords:** Cancer genomics, Mesothelioma

## Abstract

**Background:**

Multicystic mesothelioma (MCM) is a rare disease and there is debate about it’s neoplastic nature with a spectrum of disease behaviour and little known about the genomic profile. In contrast, the genomic profile of malignant peritoneal mesothelioma (MPeM) is characterised.

**Methods:**

We characterized 24 MCM and 18 MPeM cases across a panel of cancer related regions and expanded to whole-exome sequencing for 11 MCMs. Validation by amplicon sequencing and functional assessment by molecular dynamic simulation were carried out. Kaplan-Meier analysis was carried out to assess recurrence-free survival.

**Results:**

Few mutations were identified in MCMs across the panel. Exome sequencing revealed 28 genes mutated in >1 MCM case. We saw significant overrepresentation of mutations in the cohesin complex in *SMC3*, *SMC1A*, and *STAG3*. Multiple mutations in *SMC3* at codon p.E1144 indicated a mutational hotspot. Molecular dynamics simulations showed mutation at this site impacts the protein function. Amplicon sequencing confirmed hotspot mutations in further MCMs. We observed a significant association (*p* = 0.0302) of mutation in *SMC3* or *SMC1A* with disease recurrence.

**Conclusions:**

We see recurrent somatic mutations in MCMs particularly at a novel mutational hotspot in *SMC3*, consistent with a neoplastic process. Mutations in cohesin complex genes are associated with disease recurrence.

## Introduction

Multicystic mesothelioma (MCM) is an uncommon tumour arising from mesothelial cells [1–6]. It is almost always encountered in the peritoneum, where it accounts for about 3–5% of mesothelial tumours, but rare cases arising from the pleura have been described [7]. It can occur in both sexes and at any age, although women of child-bearing age are most often affected. The commonest locations are the lower abdomen and pelvis. Although many patients are asymptomatic, patients may complain of severe episodic pain, abdominal distention, urinary disturbances or constipation. Unlike other types of mesothelial neoplasm, the link between asbestos exposure and development of MCM remains uncertain.

Macroscopically, the lesions are characterised by multiple thin-walled cysts of varying size, sometimes forming confluent masses 20 cm or more in diameter. Microscopy shows the cysts to have fibrous walls lined by cells exhibiting the histological and ultrastructural features of mesothelium [1, 3, 8, 9]. Treatment can include cytoreductive surgery and hyperthermic intraperitoneal chemotherapy in selected patients with good long-term outcomes [2, 8, 10].

The nature of multicystic mesothelioma has been controversial [6]. There is evidence that it is a true neoplasm: lesions can be progressive and recur after surgery, and in some cases the lesions are associated with well differentiated papillary mesothelial tumour or adenomatoid tumour [1–3, 11, 12]. Furthermore, acquired clonal chromosome abnormalities with fusion transcripts have been demonstrated [13]. In contrast, some authors have suggested the lesions are not neoplastic and consider them a reaction to irritation following surgery, endometriosis or other causes of chronic inflammation. However, although a history of previous surgery or conditions such as pelvic inflammatory disease have been described in several patients in some series, others have not found these associations to be common [2]. Lymph node involvement was reported in one case of multicystic mesothelioma, although the pathological significance of this finding is uncertain [14].

In contrast to malignant mesothelioma (MPeM), where mutations in BAP1, NF2, CDKN2A/B and TP53 are frequently mutated or lost [15], there is little known about genetic changes in MCMs. The aim of this study was to investigate the genomic landscape of a cohort of patients with peritoneal mesotheliomas, including a focus on MCM cases and a comparison with MPeM.

## Materials and methods

### Patient sample collection

Patients with MCM or MPeM referred for consideration of cytoreductive surgery (CRS) and hyperthermic intraperitoneal chemotherapy (HIPEC) at the Peritoneal Malignancy Institute, Basingstoke, UK, were eligible for the study. Ethical approval was provided by the National Research Ethics Service, reference 09/H0504/3. All patients provided informed consent.

Histological examination of all specimens was performed by a pathologist with a specialist expertise in peritoneal pathology and classified according to standard criteria [6, 16]. Patients with MCM and malignant mesothelioma were included in the study. Any MCM lesions with areas resembling well differentiated papillary mesothelial tumour were included, provided the predominant component was histologically typical of MCM. Otherwise, lesions with features of well differentiated papillary mesothelial tumour were excluded.

Fresh tissue samples were taken intra-operatively, snap-frozen and stored in liquid nitrogen until further processing. For some patients, intra-operative samples were unavailable and material was obtained from archived paraffin wax blocks.

Clinical details were retrieved from a prospectively-maintained database with reference to clinical records as required. The presence of any residual disease at the end of the operation was recorded by the surgeon using the completeness of cytoreduction (CC) score: no visible disease (CC0), nodules of residual tumour less than 2.5 mm diameter (CC1), nodules 2.5 mm to 25 mm diameter (CC2) and nodules more than 25 mm diameter (CC3).

### DNA extraction, panel design, library preparation and sequencing

Where DNA was extracted from fresh frozen samples, samples were firstly homogenised using a Precellys 24 in 2 mL CKMix tissue homogenising tubes (Bertin Technologies SAS) at 5000 rpm for two 10 s periods. DNA was then extracted using the QIAamp DNA Blood Mini kit, automated using the QIASymphony platform (Qiagen) and DNA samples were quantified using the Qubit Fluorimeter (Life Technologies). Where DNA was extracted from paraffin embedded material, 20 µm sections were cut using a Thermo Microm HM325 microtome. Tissue was deparaffinised using Qiagen deparaffinisation solution and digested overnight using Qiagen ATL buffer and proteinase K until the tissue was fully digested. Manual extraction was then performed using Qiagen QIAamp DNA FFPE extraction kit via centrifugation. DNA was then quantified using Thermo Qubit dsDNA BR kit on a Qubit v4.0.

A custom targeted panel was designed for peritoneal malignancies including 207 key regions across 50 genes frequently mutated in cancers, with the addition of the coding regions of six genes of interest in peritoneal malignancy (*BAP1*, *CDC42*, *NF2*, *RNF43*, *SETDB1* and *TRAF7*), (Supplementary Table 1) [17]. In addition, 24 SNPs were included to enable verification of sample identity following sequencing [18]. The custom panel was generated using an amplicon-based approach (YouSeq, Winchester, UK). Capture and library preparation was performed in accordance with manufacturer’s instructions. Libraries were paired-end sequenced (300cycles) on a NextSeq500. Parallel genotyping of the 24 sample tracking panel was performed using KASP genotyping (LGC Genomics, UK).

For a subset of MCM samples, whole-exome sequencing was performed. Exome capture was undertaken using the Agilent SureSelect v6 capture kit, and libraries sequenced using a NovaSeq 6000, with a target of 24 gigabases of paired-end 150 bp reads per sample (NovoGene, HK).

To validate our findings in *SMC3*, an amplicon approach was designed to capture any mutation affecting codon 1144 of *SMC3* in a cohort consisting of 34 peritoneal mesothelioma cases, including MCM (*n* = 14) and MPeM cases (*n* = 20). The region of interest (chr10:110602904-110603018) was PCR amplified using target specific primer sequences (CTCTGATTTTTGCCATTCAGA, and TGTACTGAAACTGTACCTGAC). Primers were tagged with standard Illumina P5 and P7 adaptor sequences and sequenced on an Illumina MiSeq using a Micro kit.

### Bioinformatic and statistical analyses

For both the targeted and exome data raw sequencing fastq files were aligned to the hg38 (release 13) genome using the BWA-MEM module from the Burrows-Wheeler Aligner (BWA) software (v.0.7.17), then sorted and indexed using samtools (v.1.16.1). The sorted .bams were trimmed using the bamutil (v1.0.14) filter programme to trim ends of reads where there were >10% mismatches from the reference genome and exclude reads where there were mismatches with a cumulative phred scaled quality of 60.

For the targeted data, the trimmed bam files were input into Stitcher (v5.2.9) to merge read pairs followed by variant calling and variant quality score recalibration using Pisces (v5.2.9). For the exome data, trimmed bam files had duplicates marked (picard v2.18.14), read pairs were then merged using the bamutil clipOverlap and variants were called using mutect2 (GATK v4.1.9.0) in tumour-only mode using a panel of normal variation derived from gnomAD V2. Both the cross-sample contamination estimates and read orientation bias models were implemented to filter for erroneous variants, resulting in a refined somatic variant list. Variant quality scores were recalibrated using VariantRecalibrator (GATK v.4.1.9.0). These variants were then annotated by the Functotator software (v.4.2.2) using base data sources and output as maf format files which were analysed in maftools (v2.18.0), in Rstudio (R v4.3.2). Pathogenicity scores, including SIFT, PolyPhen2, and CADD scores were generated.

For the targeted data, variants were filtered for quality score >100 and variant allele frequency (VAF) >=2.5% (1% for *GNAS/KRAS*). For the exome data, variants were filtered for VAF > 2.5%, depth >10, and alternative allele reads >2. To exclude known germline variants for both the targeted and exome data, all variants were filtered to remove those present in gnomAD at a minor allele frequency > 0.0001. Manual curation of all mutated genes was carried out using IGV (v. 2.14.1) and mutations were assessed and tagged for exclusion based on a published standard operating procedure [19].

Overrepresentation analysis was carried out on the results of the MCM exome analysis, including the list of genes (*n* = 28) mutated in >2 samples in the exome data. Overrepresentation analysis was carried out using webGestalt (WEB-based GEne SeT AnaLysis Toolkit) [20], using the gene ontology database and biological process terms. A Fisher’s exact test was applied, and *p* values corrected by FDR.

Bioinformatic analysis of the amplicon data was undertaken using a custom pipeline able to detect variants at low alternative allele frequency as follows. The FASTQ files were aligned using BWA (v 0.7.18). Primer regions were soft-clipped using a custom in-house application, preventing variant calling in primer regions, and additionally identifying and excluding low-quality reads. Indel realignment was performed using RealignerTargetCreator and IndelRealigner from GATK (v3). Variant calling was conducted using Pisces (v5.2.10.49). Annotation was performed using a combination of well-established tools and custom in-house developed methods to generate a vcf file of results at the target region, identifying false-positive variants based on error patterns generated by the sequencing platform.

Statistical analysis of the clinical data was conducted using RStudio (R v4.3.2), to conduct Fisher’s exact tests for comparison of categorical data, and independent t-tests for continuous data. Survival analysis was conducted, using time in months from first surgery to computerised tomography (CT) evidence of recurrence, and Kaplan-Meier plots generated using the survival (v3.5–7) and survminer (v0.4.9) R packages.

### Protein modelling

Molecular Dynamics. Simulations for all systems were performed through conventional molecular dynamics using Gromacs 2024.2 [21] with the Charmm36m forcefield [22]. Details of system preparation and MD simulation parameters are described in Supplementary information.

## Results

### Patients and samples

**Fig. 1:**
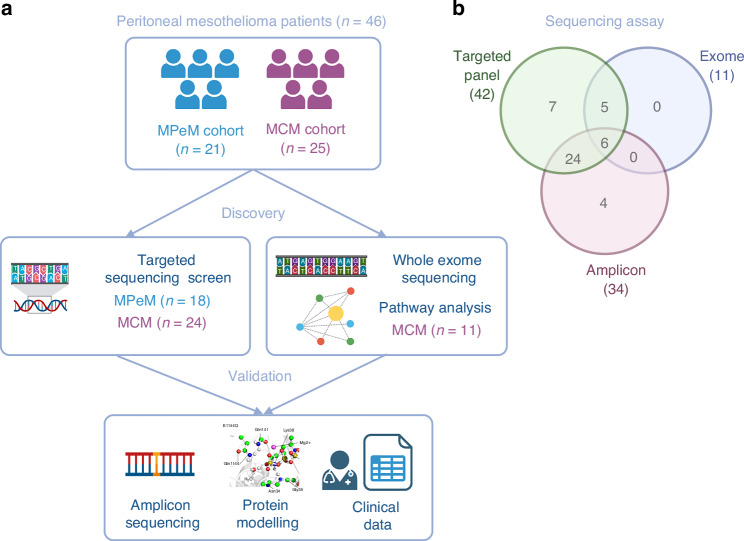
Experimental overview. **a** The mesothelioma cohorts and approaches at discovery and validation phases. **b** The overlap of cases assessed by different sequencing assays. Created in https://BioRender.com.

We collected DNA and clinical information for a total cohort of 46 patients (Fig. 1, Table 1), including 21 patients with MPeM, and 25 with MCM. There were more female cases overall (60.9%), with the preponderance of females less evident in MPeM (52.4%) compared to MCM (68%) patients. MCM cases tend to be younger with a mean age of 46.1 years at surgery (*p* = 0.0012), compared to 57.8 years for the MPeM cases.

**Table 1:** Clinical characteristics.

	MPeM (*n* = 21)	MCM (*n* = 25)	Overall (*n* = 46)
**Age at surgery** (*n* = **46**)^			
Mean (SD)	57.8 (11.8)	46.1 (11.1)	51.4 (12.8)
Median [Min, Max]	59.0 [37.0, 73.0]	45.0 [25.0, 65.0]	52.0 [25.0, 73.0]
**Sex** (*n* = **46)**			
Female	11 (52.4%)	17 (68.0%)	28 (60.9%)
Male	10 (47.6%)	8 (32.0%)	18 (39.1%)
**CC score** (*n* = **44** [20,24])*			
CC0 (no disease)	5 (25.0%)	23 (95.8%)	28 (63.6%)
CC1 ( < 0.25 cm)	11 (55.0%)	1 (4.2%)	12 (27.3%)
CC2 (0.25–2.5 cm)	2 (10.0%)	0 (0%)	2 (4.5%)
CC3 ( > 2.5 cm)	2 (10.0%)	0 (0%)	2 (4.5%)
**Surgery** (*n* = **43** [21,22]**)**			
CRS	13 (61.9%)	21 (95.5%)	34 (79.1%)
Maximum debulking	7 (33.3%)	0 (0%)	7 (16.3%)
Laparoscopy and biopsy	1 (4.8%)	1 (4.5%)	2 (4.3%)
**Patient status** (*n* = **41** [16,25])*			
Alive	4 (25%)	24 (96.0%)	28 (68.3%)
Dead	12 (75%)	1 (4.0%)	13 (31.7%)

*significant Fisher’s exact test between MPeM and MCM groups, ^significant independent t-test between MPeM and MCM groups.

One MCM showed a focal micropapillary pattern; the patient is alive with no recurrence at follow-up (120 months). All other MCM patients displayed typical features of MCM throughout the lesions. There were two MPeM cases which were biphasic, while the remainder were epithelioid.

Five MCM cases had disease recurrence as reported from CT findings. The mean time from first surgery to recurrence was 56.4 months (median = 43, range 12–147 months). All but one MCM cases were alive at most recent follow-up (mean = 89.5, median = 115.8 months) (Fisher’s exact *p* = 0.0001). The MCM group has a significantly higher number of cases in the “no disease” CC score category (Table 1).

### Somatic mutations in the discovery phase

**Fig. 2:**
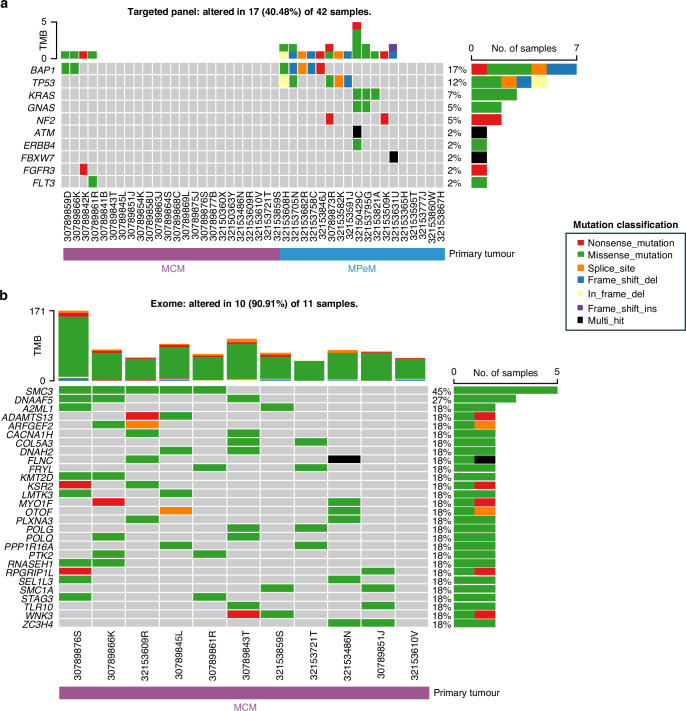
Oncoplots. Somatic mutations detected in **a** the targeted panel and, **b** exome sequencing of peritoneal mesothelioma cases.

Firstly, we present mutational data identified through sequencing of a targeted panel applied to 42 patients with peritoneal mesothelioma, including 18 MPeM and 24 MCM cases. Although the genomic targets in this panel include those regions most frequently mutated across all cancers, this discovery approach identified very few mutations present in the MCM cases. We therefore extended our discovery phase to sequencing the whole exome of 11 MCM patients (Fig. 2). The mean sequencing depth was >500X for the targeted panel and >295X for the exome data (Supplementary Table 2), indicating good coverage of targeted regions.

After filtering and manual curation, a range of mutations were detected in the targeted panel regions in the 18 MPeM cases (Fig. 2a). We observed mutations in two or more tumour samples in five genes (*BAP1*, *TP53*, *KRAS*, *GNAS* and *NF2*) with *BAP1* and *TP53* mutations being the most frequent, each detected in 5/18 (27.8%) of cases. There were two cases with both *BAP1* and *TP53* mutations. *KRAS* and *GNAS* missense mutations were seen in three and two cases respectively. *KRAS* and *GNAS* mutations occurred in cases which were not mutated for *BAP1* or *TP53*. Two cases harboured nonsense mutations in *NF2*.

Across all gene regions targeted in our cancer panel, mutations were detected in just 4/24 (16.6%) MCM tumour samples. Two cases of MCM contained mutations in *BAP1*. Both showed histological features typical of MCM. Two additional MCM cases had mutations in *FGFR3* and *FLT3*, genes not mutated in the MPeM cohort.

With 20/24 (83%) of MCM cases showing no mutations across the genes represented in the targeted panel, a set of 11 MCM cases underwent exome sequencing. Tumour mutation burden calculated from the exome data for the MCM cases was low, a median of 1.44/Mb (Supplementary Fig. 1B). After filtering and manual curation, there were 28 genes mutated in two or more samples (>10%) (Fig. 2b).

Of the MCM cases with *BAP1* mutations previously detected, exome sequencing was available for one of these patients; it showed that the lesion also harboured a mutation in *SMC3* and the mutational burden was 1.52/MB. This patient developed a pelvic recurrence of MCM and received a second CRS and HIPEC 12 months after the first surgery, and was alive and well nine years after the initial diagnosis. Unfortunately, no exome sequencing data was available for the second patient who was alive seven years after diagnosis with a single indeterminate nodule in the left lower lobe.

**Fig. 3:**
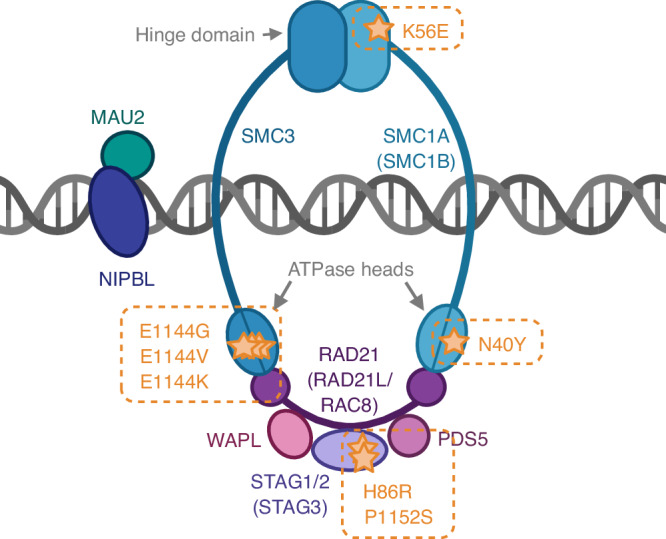
The cohesin complex. A diagram of the mitotic cohesin complex showing the somatic mutations detected by exome sequencing observed in the MCM cases. Meiosis-specific components are essential for proper chromosome behaviour during meiotic cell division, these components are shown in brackets. The codon locations are highlighted in orange in the genes *SMC3*, *SMC1A*, and *STAG3*. Created in https://BioRender.com.

From the exome data, the most frequently mutated gene, *SMC3*, was mutated in 45% (5/11) of cases and the second most frequent *DNAAF5* was observed in 27% (3/11 cases). The remaining 26 genes were mutated in two cases each (2/11 = 18%) (Fig. 2b). Overrepresentation analysis was conducted including all 28 genes that were mutated in >1 sample. GO biological process terms were analysed as the gene set and the related terms ‘establishment of sister chromatid cohesion’ (GO:0034085) and ‘meiotic sister chromatid cohesion’ (GO:0051177) were significantly overrepresented after FDR correction (*p* = 0.003 and 0.005, respectively) (Supplementary Table 3). Both terms highlighted three genes, the most frequently mutated gene *SMC3*, along with *SMC1A* and *STAG3* all related to the cohesin complex (Fig. 3). The mutations in *SMC3* and *SMC1A* are mutually exclusive, however mutations in *SMC3* and *STAG3* are co-occurring (Fig. 2b). The two *STAG3* mutations were not predicted to be deleterious and the p.H86R mutation has been seen as a rare variant in the population (gnomAD allele frequency = 5.89e–5) (Supplementary Table 4). The *SMC3* mutations occur at the same codon (p.E1144) in the ATPase domain of the protein indicating a potential mutational hotspot. In our modestly sized cohort, we observe 3 different nucleotide changes affecting this codon, and all occur in the MCM cases at low variant allele frequencies (VAF = 4.2–9.7%) (Fig. 3, Supplementary Fig. 2A). The mutations detected in *SMC3* and *SMC1A* were absent from the gnomAD database and predicted to be deleterious to function using a range of pathogenicity scores (Supplementary Table 4).

### Validation of somatic mutations in SMC3

**Fig. 4:**
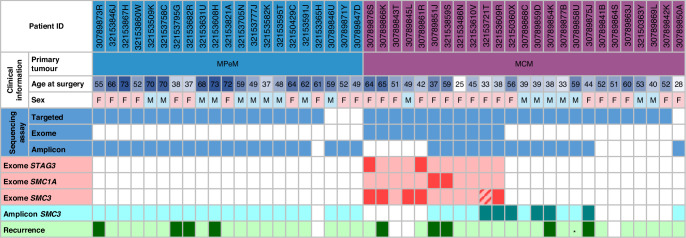
Matrix showing clinical information, assay type, and cohesin mutations across MPeM and MCM cases. The pink/red hashed cell represents a somatic mutation that was present when visualising the exome data using the IGV software, but was not called by the variant calling software due to low variant allele frequency. *represents a patient that has died without recurrence. For the exome and amplicon genetic observations across *STAG3*, *SMC1A* and *SMC3* genes, the darker shade indicates presence of a mutation and the lighter shade indicates no mutation was identified. Blank/white cells indicate the patient’s tumour sample was not tested.

Following identification of a mutational hotspot in *SMC3* we carried out amplicon sequencing of the p.E1144 codon in exon 27 of *SMC3* in order to validate our findings in 34 samples with available DNA, including MPeM (*n* = 20) and MCM cases (*n* = 14). Of the six MCM cases with both exome and amplicon data, amplicon sequencing confirmed the absence of *SMC3* mutations in four cases, and the presence of an *SMC3* mutation in one case. In the sixth case amplicon sequencing detected an additional low VAF *SMC3* mutation (VAF = 1.8%) which had not been called in the exome data. This mutation was subsequently confirmed present at very low variant allele frequency in the exome data by visualisation of raw NGS reads in IGV (VAF = 1.7%, 4/235 reads), but had failed the required quality threshold for variant calling in exome data. In MCM cases without previous exome data (*n* = 8), we identified a further four mutations at this codon (Fig. 4). There was no evidence for any p.E1144 mutations detected across any of the 20 MPeM cases tested.

Across both exome and amplicon assays, we found a total of 10/19 (52.6%) MCM cases tested had mutations in codon E1144 of *SMC3*. These mutations result in changes of the Glutamic acid (E) to lysine (K), valine (V) and glycine (G) (*n* = 4, 4, and 2, respectively) (Fig. 3, supplementary Table 4).

### Protein modelling

**Fig. 5:**
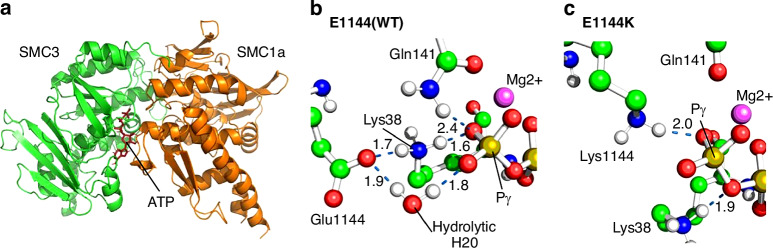
Molecular dynamics simulations results. **a** Cartoon representation of the SMC3-SMC1a-ATP ATPase domain simulation setup. **b** Interaction network around the γ-phosphorus atom and mutant residue 1144 sidechain at 200 ns simulation time for the WT system. **c** Interaction network for the E1144K mutant, notably lacking a water at the putative binding site. Relevant hydrogen bonds are noted where present with a dashed blue line and distance in angstroms labelled.

To model the functional effects of the SMC3 mutations, we performed conventional molecular dynamics simulation of the SMC3/SMC1a ATP binding domain with ATP bound to SMC3 (Fig. 5a) for the wild-type (E1144) and four loss of function mutants (E1144K, E1144V, E1144G and E1144Q) [23]. Three of these mutants are identified in this work (E1144K, E1144V and E1144G), while E1144Q has been identified as a knock-out mutation for cryo-EM [23]. E1144 was found to uniquely coordinate a water molecule in the 2–4 Å gap between itself and the terminal ATP Pγ atom (Fig. 5b), expected to facilitate ATP hydrolysis. This is highly similar to the interaction network formed by the equivalent residue, E1157, in SMC1a [24]. All four simulated mutants disrupt this interaction by altering the location of the hydrolytic water molecule site (Supplementary Fig. 2A), either through direct occlusion of the site as in E1144K (Fig. 5c), E1144V and E1144Q, or through the inability to contact and stabilise a water at this position (E1144G). A detailed account and depiction of the individual simulation results are available in the supplementary material.

### Survival analysis

All five MCM cases with disease recurrence carried a mutation in either *SMC3* or *SMC1A* (Fig. 4). Kaplan-Meier survival analysis showed a significant increase in disease recurrence (*p* = 0.0302) in the mutated cases (Supplementary Fig. 3) indicating clinical relevance of mutations in the cohesin complex in disease recurrence.

## Discussion

In this study, we applied a discovery and validation approach to survey the genomic landscape of two rare peritoneal malignancies. Our findings indicate the genomic architecture of MPeM and MCM peritoneal mesotheliomas are markedly different. The MPeMs harboured relatively numerous mutations within known cancer hoptspot regions, generally consistent with those found in previous studies, including *BAP1, TP53* and *NF2*. Although, the proportion of MPeMs with *BAP1* mutation was lower in our case series (5/18, 28%) than reported previously [25, 26]. This relative deficit may be partly attributable to our limited focus on single nucleotide variants only - *BAP1* copy number changes are also known to be important but are not accurately detected by technologies applied in this study.

We identified *KRAS* mutations in 3/18 (16%) of MPeMs. This figure is higher than the 1.4% reported by Hitbrunner et al. [15]. Our observation of mutations in *ATM* and *FBXW7* have also been previously reported [15]. We detected *GNAS* mutations in 2/18 (11%), and an ERBB4 mutation in 1/18 (5.5%), neither of which have been previously noted in the literature in MPeM.

The targeted panel revealed markedly fewer mutations in the MCMs indicating genetic changes in multicystic mesothelioma do not generally fall within known cancer hotspots. Two lesions (2/24, 8%) showed *BAP1* mutation, but there were no discernible differences in clinical or histological features between these and the other 22 cases of MCM. However, exome sequencing of a subset of 11 patients revealed 28 other genes that were mutated in at least two samples. Of particular note, the expanded exome data showed recurrent mutations in cohesin subunit genes in 7/11 (64%) patients. *SMC3* was mutated in 5/11 (45%), *SMC1A* in 2/11 (18%) and *STAG3* in 2/11 (18%).

Of the two mutations in *STAG3*, one had been seen previously in gnomAD, although very rare, but neither were predicted to be deleterious by in silico prediction tools. These data suggest the evidence for a driver role of the mutations in *STAG3* is relatively weak. However, the mutually exclusive mutations in *SMC1A* and *SMC3* were all predicted to be highly deleterious. *SMC3* and *SMC1A* have been observed as somatically mutated in a number of cancers previously, including acute myeloid leukaemia [27], bladder cancer [28], and colon cancer [29]. Overexpression of *SMC3* has been shown to cause transformation of mammalian cells [30]. Expression of *SMC3* across healthy tissues shows high expression in testis, brain, ovary and uterus, while expression of SMC1A is high in uterus, brain, and cervix [31]. Along with other cohesin component genes such a *NIBPL*, germline mutations in both *SMC3* (hinge-domain) and *SMC1A* (hinge and head-domains) have been observed to cause Cornelia de Lange syndrome (CdLS). These variants contribute to ∼5% of cases and result in a consistently mild phenotype [32].

*SMC3* is most frequently mutated in endometrial cancer in the cancer genome atlas (TCGA) data, with (45/512) 8.8% of cases carrying a mutation. The most common mutations are a frameshift deletion (Y43Mfs*69) and stop gain (R99*), both present in 4 samples (0.8%) (GDC, Data Release 42.0–January 30, 2025) [33]. In our MCM cases, using exome and amplicon sequencing we have identified a mutation hotspot at p.E1144 mutated in 53% (10/19) of cases; interestingly, there are no mutations at this codon within the ATPase domain in the TCGA dataset.

Mutations at the SMC3 E1144 residue are known to disrupt ATPase activity [34]. However, the absence of a crystal structure for the wild-type active conformation of SMC3 has limited our understanding of how these mutations impair activity. E1144 is believed to coordinate a water molecule that mediates the hydrolytic attack of the β-γ phosphodiester bond, with similar conserved glutamic acids having been observed in high resolution crystal structures of numerous ATPases e.g. BmrA [35], MJ0796 [36] and F1 ATP synthase [37]. The functional catalytic role of these glutamate residues, structurally equivalent to E1144, has also been modelled in yeast SMC1a using QM/MM simulations of enzyme ATP hydrolysis [24]. Our molecular dynamics simulation results, consistent with similar ATPase mechanisms previously identified in the literature, support the view that E1144 mutations may disrupt water molecule coordination with ATP, leading to disruption of ATPase function in SMC3.

The cohesin multiprotein complex organizes the eukaryotic genome and is required for sister chromatid cohesion. Cohesin has a range of functions throughout the cell cycle and ATPase activity mediated by the *SMC3* and *SMC1A* ATPase domains is central to cohesin’s ability to dynamically interact with DNA to perform these functions [38]. Somatic mutations in cohesin complex genes are thought to contribute to cancer development through multiple mechanisms. The cohesin complex plays an important role in sister chromatid cohesion, and thus mutations affecting this function are thought to contribute to cancer by inducing genome instability, aneuploidy, and altered DNA replication. However, further research is necessary understand the mechanism(s) by which cohesin mutations drive neoplastic transformation [39]. In interphase, cohesin has a crucial role in genome organisation by determining chromatin structure and thus regulating gene expression through DNA looping [39]. There is evidence of mutations in cohesin genes including *SMC1A* causing changes to gene expression which may affect cell identity, differentiation, and proliferation [40]. A deeper understanding of how mutations in cohesin affect these processes in cancer could contribute the development of novel therapeutic strategies [39].

This study is the first to show recurrent mutations in MCM consistent with a neoplastic phenotype. We also found that mutations in cohesin complex genes *SMC3* or *SMC1A* are associated with an increased risk of recurrence in patients referred for CRS and HIPEC. Although a modest sample size, in this study all 5 of the MCM cases with recurrence had a mutation in one of these two genes. Furthermore, our findings increase the number of tumour types associated with mutations in cohesin complex genes. Further investigation in additional MCM cases, along with correlations to common clinical parameters, characterization of complex lesions, and extended follow-up of identified cases, may strengthen our findings. These efforts could support the potential utility of cohesin gene analysis as both a diagnostic tool and a biomarker for recurrence.

## Conclusion

The finding of recurrent mutations in MCM is consistent with it being a true neoplasm, not a reactive process. Cohesin complex genes appear to be involved in pathogenesis, with mutations in *SMC3* found in 10/19 (53%) and *SMC1A* in 2/11 (18%) of samples analysed in a mutually exclusive manner. We have identified a functionally relevant mutation hotspot in *SMC3* at codon 1144, and show that the risk of disease recurrence is higher if lesions contain mutations in either *SMC3* or *SMC1A*.

## Supplementary information

Supplementary material

Supplementary table 1

Supplementary table 2

Supplementary table 3

Supplementary table 4

## Data Availability

The data that support the findings of this study are not openly available due to reasons of sensitivity and are available from the corresponding author upon reasonable request.
