# Supplementary material for: Genomic sequencing of multicystic mesothelioma finds cohesin complex mutations associated with disease recurrence in patients referred for cytoreductive surgery and HIPEC
Source: Br J Cancer. 2026 Mar 14;134(9):1352–9. doi: 10.1038/s41416-026-03366-5 (PMC13079845; doi:10.1038/s41416-026-03366-5)

**Supplementary material**

*Supplementary Methods*

The SMC3-SMC1 ATPase domain derived from a cohesin cryo-EM structure (6WGE, 3.9 Å) [1] was protonated using ProPKa3.1 [2] to a pH of 7. ANP bound to SMC1a was removed from the structure and the SMC3 ANP was mutated to ATP. Mutations were introduced using PyMol 2.5.2 mutagenesis tool. The system was solvated in a cubic box of CHARMM-TIP3P waters using a 1.2 nm edge distance and neutralised to 150 mM ionic concentration with Na+ and Cl- ions. Energy minimisation was initially performed using steepest descent with 5000 steps and step size of 0.01 nm. NVT equilibration was performed for 100 ps using the Bussi-Donadio-Parrinello thermostat [3] at 310 K at 0.1 ps time constant. NPT equilibration was subsequently performed for an additional 100 ps using the Berendsen barostat [4] with 1 bar reference pressure at 2.0 ps time constant. Each simulation replica began from the NPT equilibrated final step. Production runs were performed in the NPT canonical ensemble using the Bussi-Donadio-Parrinello thermostat with 0.1 ps time constant and 310 K and Parrinello-Rahman barostat [5] with 2.0 ps time constant and 1 bar reference pressure. Long-range electrostatics were calculated using particle mesh Ewald (PME) with a short-range real space summation cut-off at 1.2 nm. Lennard-Jones interactions were similarly cut off at 1.2 nm. A LJ force-switch function was applied between 1.1-1.2 nm. Production simulations were performed for 200 ns with three repeats per variant from the same starting structure with randomised velocities. Radial distribution functions were calculated using the MDAnalysis v2.0 [6] InterRDF function for all heavy atoms of the indicated sidechain.

*Supplementary Results*

A radial distribution function was plotted to visualise the positioning of the residue 1144 sidechain around the γ-phosphate for each variant (**Supplementary Figure 2A**) across all concatenated simulation data. We observed that E1144Q, E1144K and E1144V formed close contacts to the γ-phosphorus atom, compared to E1144 which retained a 2 Å gap, sufficient for a water molecule to lie between the sidechain and terminal γ-phosphate group. The final simulation frame at 200 ns for repeat 1 of each simulation was visualised to explore the functional effect of each mutation. E1144 was observed to coordinate a water molecular adjacent to the γ-phosphate, whilst N-containing groups from Gln141, Lys38, Asn34 and Gly35 coordinated the surrounding γ and β-phosphate oxygen atoms (**Figure 5B and Supplementary Figure 2B**). QM/MM models of ATP hydrolysis support the mechanism whereby a proton is abstracted by deprotonated E1144 from the adjacent water molecule to generate a hydroxide anion, which in turn hydrolyses the adjacent ATP γ-phosphate [7]. In all the mutant simulations, we observe no negatively charged sidechains replacing the E1144 interaction with water, meaning that the hydroxide ion intermediate cannot be generated or stabilised (**Supplementary Figure 2C-F**). Only amide groups establish productive contacts to Pγ and nearby waters, which are presumably incapable of abstracting a proton and stabilising the resulting negative hydroxide ion intermediate, when compared with the negative glutamate sidechain. The functional impact of E1144V was less clear as the primary N-containing network previously identified as stabilising the γ and β-phosphate oxygen atoms was retained (**Supplementary Figure 2E**), though some direct occlusion of the water binding position was observed in the simulation, and no sidechains coordinated the putative hydrolytic waters (**Supplementary Figure 2A/D**). As such the hydrolysis of ATP is likely impaired by the lack of direct coordination of the hydrolytic water. The E1144G mutation causes significant disruption to the local N-containing γ-phosphate support network with significant occlusion of the canonical water binding site through reorientation of the Lys38 residue (**Supplementary Figure 2F**). Regardless of exact local changes, all mutants fail to recreate either a coordinating interaction of a water molecule with surrounding amide support structure, or replace the capability of E1144 to generate the negatively charged hydroxyl intermediate necessary for ATP hydrolysis, in support of their loss-of-function effect.

*Supplementary References*

1. Shi, Z., et al., Cryo-EM structure of the human cohesin-NIPBL-DNA complex. Science, 2020. 368(6498): p. 1454-1459.

2. Chresten R. Søndergaard, Mats H. M. Olsson, Michał Rostkowski, and Jan H. Jensen. Improved Treatment of Ligands and Coupling Effects in Empirical Calculation and Rationalization of pKa Values. Journal of Chemical Theory and Computation 2011 7 (7), 2284-2295. DOI: 10.1021/ct200133y

3. Giovanni Bussi, Davide Donadio, Michele Parrinello; Canonical sampling through velocity rescaling. J. Chem. Phys. 7 January 2007; 126 (1): 014101. https://doi.org/10.1063/1.2408420

4. H. J. C. Berendsen, J. P. M. Postma, W. F. van Gunsteren, A. DiNola, J. R. Haak; Molecular dynamics with coupling to an external bath. J. Chem. Phys. 15 October 1984; 81 (8): 3684–3690. https://doi.org/10.1063/1.448118

5. M. Parrinello, A. Rahman. Crystal Structure and Pair Potentials: A Molecular-Dynamics Study. Phys. Rev. Lett. 45, 1196 – Published 6 October, 1980. DOI: https://doi.org/10.1103/PhysRevLett.45.1196

6. Michaud-Agrawal, N., Denning, E.J., Woolf, T.B. and Beckstein, O. (2011), MDAnalysis: A toolkit for the analysis of molecular dynamics simulations. J. Comput. Chem., 32: 2319-2327. https://doi.org/10.1002/jcc.21787

7. Marcos-Alcalde, I., et al., *Two-step ATP-driven opening of cohesin head.* Sci Rep, 2017. **7**(1): p. 3266.

**Supplementary Tables.** Provided as an MS excel file.

**Supplementary table 1.** Sequencing panel targets.

**Supplementary table 2.** Sequencing coverage statistics (A) Targeted panel, (B) Exomes.

**Supplementary table 3.** Overrepresentation analysis.

**Supplementary table 4.** Somatic mutations in cohesin related genes detected in MCM cases.

**Supplementary Figure 1**. (A) Variant allele frequencies (y-axis) for the genes with 2 or more somatic mutations detected in the MCM exome data. (B) Mutational burden MCM exome data (n=11). TMB/MB = tumour mutation burden per megabase.


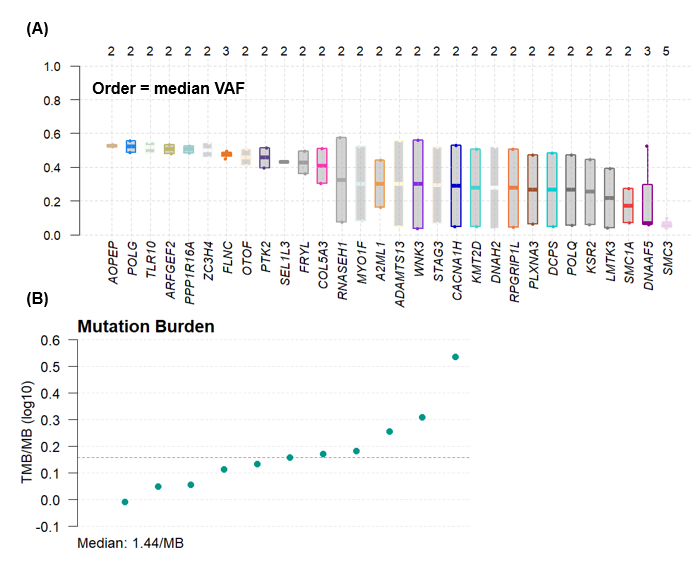


**Supplementary Figure 2.** (A) Residue 1144 side chain heavy atom distribution relative to its distance from the Py atom described through a radial distribution function across all three simulation repeats. (B-F) Interaction networks around γ-phosphorus atom at 200 ns simulation time for (B) Wild-type (C) Q1144 (D) K1144 (E) V1144 and (F) G1144. Relevant coordinating waters between 1144 and γ-phosphate have been shown where observed.


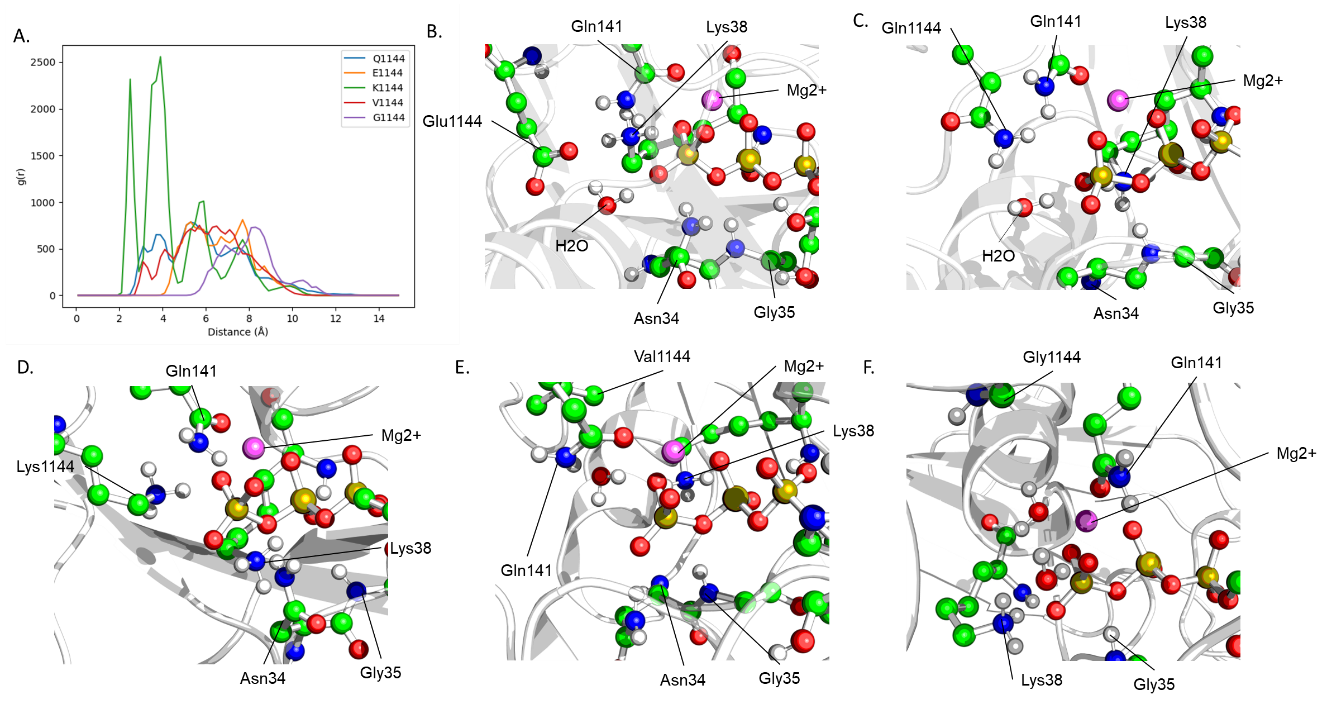


**Supplementary Figure 3.** Kaplan-Meier survival curve showing an increase in disease recurrence in cases harbouring mutations in either *SMC3* or *SMC1A*. Event is recurrence. Time in months from first surgery to recurrence reported from CT findings.


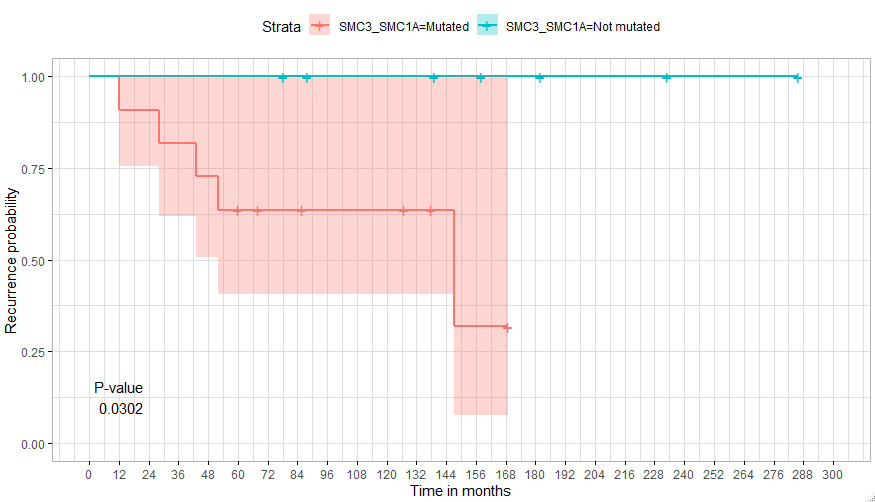

Supplement: Supplementary file 1 — Supplementary material [file 41416_2026_3366_MOESM1_ESM.docx]
